# Reward and punishment sensitivity and disordered eating behaviors in men and women

**DOI:** 10.1186/s40337-017-0138-2

**Published:** 2017-02-16

**Authors:** Kalina T. Eneva, Susan Murray, Jared O’Garro-Moore, Angelina Yiu, Lauren B. Alloy, Nicole M. Avena, Eunice Y. Chen

**Affiliations:** 1 Temple University, 1701 N. 13th St., Philadelphia, PA 19147 USA; 2 Department of Pharmacology and Systems Therapeutics, Mount Sinai School of Medicine, One Gustave L. Levy Place, Box 1677, New York, NY 10029 USA

**Keywords:** Eating disorders, Reward sensitivity, Punishment sensitivity, Gender differences

## Abstract

**Background:**

Reward and punishment sensitivities have been identified as potential contributors to binge eating and compensatory behaviors, though few studies have examined gender differences in these behaviors.

**Method:**

A college-aged sample (*N* = 1,022) completed both the Eating Disorders Diagnostic Scale (EDDS) and Sensitivity to Punishment/Sensitivity to Reward Questionnaire (SPSRQ).

**Results:**

Rates of binge eating were similar in males and females. Among those reporting compensatory behaviors, women reported engaging in compensatory behaviors more frequently than men. Sensitivity to reward and sensitivity to punishment were both positively associated with binge eating frequency in both genders. In contrast, women with high reward sensitivity reported engaging in compensatory behaviors more frequently.

**Conclusions:**

Rates of binge eating and compensatory weight control behaviors were similar between college-aged males and females, though females who engaged in compensatory behaviors did so more frequently than males. Sensitivity to punishment was greater in females, whereas sensitivity to reward was greater in males. Reward and punishment sensitivity were each positively associated with binge eating in both males and females, while only reward sensitivity was positively associated with compensatory behaviors in females.

## Plain English Summary

The current study explored associations between reward and punishment sensitivity and key behavioral features of eating disorders in women and men. This study demonstrates significant associations between high reward and punishment sensitivities and specific eating disorder behaviors in both males and females. Specifically, both reward and punishment sensitivity were positively associated with binge eating in males and females. Further, high sensitivity to reward was positively associated with compensatory behaviors among women. Additional research aimed at further understanding the role of reward and punishment sensitivity in the development and maintenance of eating disorders is needed.

## Highlights

- Rates of binge eating and compensatory weight control behaviors were similar between college-aged males and females.
- Females who engaged in compensatory behaviors did so more frequently than males who engaged in compensatory behaviors.
- Sensitivity to punishment was greater in females, whereas sensitivity to reward was greater in males.
- Reward and punishment sensitivity were each positively associated with binge eating in both males and females.
- Reward sensitivity was positively associated with compensatory behaviors in females.

## Background

Heightened reward sensitivity, a measure of an individual’s tendency to seek out and experience pleasure from positive reinforcement, is proposed as a vulnerability for Binge Eating Disorder and Bulimia Nervosa [1–3], eating disorders that affect approximately 3.6% of individuals in the United States [4]. Heightened reward sensitivity may increase the salience of food taste and promote overeating and is proposed to be part of the impulsivity construct [5–10]. In addition to aberrant reward sensitivity, individuals across the range of eating disorders show heightened punishment sensitivity, defined by the avoidance of negative responses and consequences [2, 11–13]. Associations between punishment sensitivity and symptoms of bulimia have prompted the hypothesis that higher punishment sensitivity may be related to an increased fear of being overweight which may lead to greater weight control behaviors [14]. Taken together, it is postulated that the binge-purge cycle is perpetuated in part by greater reward sensitivity driving binge eating and greater punishment sensitivity contributing to compensatory weight control behaviors (e.g., fasting, laxative use, diuretic use, vomiting).

Reward sensitivity and punishment sensitivity have also been shown to differ by gender. A meta-analysis found that relative to men, women tend to report greater reward dependence, or the tendency to respond intensely to signals of reward [15], whereas men report significantly higher sensation seeking, or the tendency to seek experiences and feelings that are novel, pleasurable and intense [16]. Further, women demonstrate greater punishment sensitivity compared to men [16]. The majority of studies examining associations between reward and punishment sensitivities and eating disorders have included only female clinical samples. However, 7.5% of men in a large non-clinical sample reported binge eating [17], suggesting that further research aimed at understanding specific factors that may motivate binge eating, such as reward and punishment sensitivity, in men is warranted.

We first examined and compared the prevalence of binge eating and compensatory behaviors in each gender among a sample of college students. We hypothesized that women would be more likely to engage in binge eating and compensatory behaviors and would report greater sensitivity to both reward and punishment than men [15, 16]. Further, we predicted that reward sensitivity is positively associated with binge eating [5] and that punishment sensitivity is positively associated with compensatory behaviors [14].

## Methods

### Participants

Participants (*N* = 1,022) were undergraduate students at a large Northeast mid-Atlantic university enrolled in introductory-level psychology courses. Participants completed a battery of self-report questionnaires as a component of class participation. Demographic information, including gender, age, race and ethnicity, was collected. Participants were excluded if they were below 18 years of age and if they elected to discontinue their participation in the study. All study procedures were approved by the University’s Institutional Review Board.

### Measures

Sensitivities to reward and punishment were assessed using the Sensitivity to Punishment/Sensitivity to Reward Questionnaire (SPSRQ) [18]. The SPSRQ includes 24 items that assess reward sensitivity (e.g., “Do you often do things to be praised?”) and 24 items that assess punishment sensitivity (e.g. “Do you often refrain from doing something because you are afraid of it being illegal?”). In the current study, the SPSRQ demonstrated an internal consistency of α = .90 for the reward sensitivity subscale and α = .86 for the punishment sensitivity subscale. Individuals endorse or deny each item, and total scores for sensitivity to reward and sensitivity to punishment range from 0 to 24. Binge eating and compensatory behaviors were assessed using the Eating Disorders Diagnostic Scale (EDDS), a 22-item questionnaire which has been validated in individuals with and without eating disorders [19]. The EDDS assesses frequency of binge eating by asking individuals to report the number of times per week (16 options: 0 to 14 times inclusive, and ‘over 14 times’) they ate an unusually large amount of food while experiencing a sense of loss of control over the past three months. Using the same scale, compensatory behaviors were assessed by asking individuals to report the number of times per week they engaged in compensatory behaviors over the past three months.

### Data analytic plan

A chi-square analysis was conducted to examine gender differences in the engagement of binge eating and compensatory behaviors. To assess gender differences in the number of episodes of binge eating and compensatory behaviors and subscale scores for reward sensitivity and punishment sensitivity, two-tailed t-tests were conducted. Pearson correlations between age, BMI, compensatory behavior, and binging behavior were examined to determine variable inclusion in a hierarchical linear regression. Associations between centered reward sensitivity and centered punishment sensitivity and frequency of binge eating and compensatory behaviors were examined with four hierarchical linear regressions. Binge eating or compensatory behavior frequency served as dependent variables. Gender, centered values of reward sensitivity or punishment sensitivity, and the interaction between gender and reward sensitivity or punishment sensitivity were added in hierarchical linear regressions. Results are described as significant where *p* < .05.

## Results

### Sample

The mean age of participants was 21 years and median age was 20 years (*SD* = 4). The sample was predominantly female (64.38%) and identified as Caucasian (66.7%), followed by African-American (14.9%), Asian-American (13.7%), or ‘Other’ (4.4%). The sample had a self-reported mean BMI of 23.79 kg/m2 (*SD* = 4.67). BMI was significantly associated with binge eating (*r* = .10, *p* < .01) but not compensatory behaviors (*r* = .03, *p* = .31). Age was not associated with either binge eating (*r* = .02, *p* = .58) or compensatory behaviors (*r* = −.02, *p* = .46). Therefore, only BMI was included as a covariate for binge eating.

### Prevalence of binge eating and compensatory behaviors in men and women

Table 1 presents average weekly binge eating and compensatory behavior frequencies over the past three months and SPSRQ subscale scores for men and women separately. No assumptions were violated for chi-square and *t*-test analyses (Shapiro-Wilk test *p* = .21). Binge eating was reported among a subset of the sample (*n* = 248 [23.9% women; 26.1% men]) and the prevalence of binge eating did not differ significantly by gender (*χ* ^2^(1) = 0.82, *p* = .41). Moreover, binge eating frequency did not vary by gender (*t*(137) = 0.57, *p* = .53). A similar proportion of participants (*n* = 255 [29.3% women; 24.7% men]) reported compensatory behaviors, with a trend towards a greater number of women reporting compensatory behaviors relative to men (*χ* ^2^(1) = 2.48, *p* = .07). Of those reporting compensatory behaviors, 36.2% reported excessive exercise, 33.9% reported vomiting, 15.8% reported fasting, and 14.1% reported using laxatives. Women engaged in compensatory behaviors with greater frequency than men (*t*(72) = 3.58, *p* < 0.01).

**Table 1:** Differences between males and females in eating disorder behavior frequency and to reward and punishment

	Females	Males	t(df)
	M (SD)	M (SD)	
Objective Binge Eating Frequency (EDDS) (*N* = 248)	1.43 (2.21)	1.51 (2.32)	0.44 (247)
Compensatory Behavior Frequency (EDDS) (*N* = 255)	4.10 (6.35)	3.15 (7.42)	2.32 (254)**
Punishment Sensitivity (SPSRQ) *N* = 1020	12.74 (5.73)	10.84 (5.75)	−4.84 (1018)**
Reward Sensitivity (SPSRQ) *N* = 1020	11.48 (4.75)	13.02 (5.20)	4.40 (1018)**

**p < .01

*EDDS* Eating Disorders Diagnostic Scale

*SPSRQ* Sensitivity to Punishment/Sensitivity to Reward Questionnaire

### Sensitivity to punishment and reward

Women showed significantly higher scores on the punishment sensitivity subscale compared to men (*t*(1018) = 3.21, *p* < 0.01), whereas reward sensitivity was significantly higher among men than women (*t*(1018) = 2.93, *p* < 0.01). For the group that engaged in both binge eating and compensatory behavior (*N* = 107), the mean of the punishment sensitivity subscale was *M* = 12.89 (*SD* = 5.70) and their mean reward sensitivity subscale was *M* = 12.54 (*SD* = 5.23).

### Relationship between reward and punishment sensitivity and eating disorder behavior and gender

Punishment sensitivity and reward sensitivity, but not gender, independently and significantly predicted the frequency of binge eating episodes (see Table 2). The interaction between gender and sensitivity to reward significantly predicted the frequency of compensatory behaviors, such that women showed greater frequency of compensatory behaviors at high levels of reward sensitivity. The same regression analysis was conducted on the group (*N* = 107) who engaged in both binge eating and compensatory behavior and these results did not change.

**Table 2:** Contribution of reward and punishment sensitivity to objective binge eating frequency and compensatory behavior frequency in males and females *N* = 1020

	Adjusted *R* ^2^	ΔAdjusted *R* ^2^	*b*	SE	β	*p*
Binge eating^b^						
Regression 1:						
Step 1:	0.01					.02
BMI			0.04	0.02	0.08	.02
Step 2:	0.02	0.02				.00
BMI			0.04	0.02	0.08	.03
Gender^a^			0.02	0.16	0.00	.91
Punishment sensitivity^c^			0.07	0.02	0.18	.01
Gender x Punishment Sensitivity^c^			−0.02	0.03	−0.05	.40
Regression 2:						
Step 1:	0.01					.04
BMI			0.04	0.02	0.07	.04
Step 2:	0.03	0.02				.00
BMI			0.04	0.02	0.08	.03
Gender^a^			0.20	0.16	0.04	.23
Reward sensitivity^c^			0.05	0.03	0.11	.04
Gender x Reward Sensitivity^c^			0.02	0.03	0.04	.50
Compensatory behaviors^b^						
Regression 3:	0.01					.28
Gender^a^			0.23	0.46	0.02	.62
Punishment sensitivity^c^			0.04	0.06	0.03	.57
Gender x Punishment Sensitivity^c^			0.05	0.08	0.03	.56
Regression 4:	0.01					.01
Gender^a^			0.52	0.47	0.04	.26
Reward sensitivity^c^			0.03	0.07	0.02	.69
Gender x Reward Sensitivity^c^			0.22	0.09	0.12	.02

^a^Demographic Survey; Males coded as 0, Females coded as 1

^b^Eating Disorders Diagnostic Scale (EDDS)

^c^Sensitivity to Punishment/Sensitivity to Reward Questionnaire (SPSRQ)

## Discussion

In the current study, engagement in binge eating and compensatory behaviors did not vary significantly by gender, although there was a trend towards a greater proportion of women engaging in compensatory behaviors. Men and women reported comparable weekly binge eating episodes over the past 3 months, with rates similar to those reported by previous studies in undergraduate samples of men and women [20–22]. In contrast, women reported significantly more frequent compensatory behaviors than men. Consistent with our hypothesis and previous research [15, 16], punishment sensitivity was higher among females than males. Contrary to our hypothesis, males reported greater reward sensitivity than females, although this finding is consistent with earlier research in undergraduate students [18]. Binge-eating was associated with both reward sensitivity and punishment sensitivity. Compensatory behavior was not associated with punishment sensitivity but rather with reward sensitivity in women.

Findings indicate that both reward sensitivity and punishment sensitivity were positively associated with binge eating in males and females. The current findings support research suggesting that reward sensitivity may promote binge eating [7, 23]. The finding that punishment sensitivity was also significantly related to binge eating suggests that binge eating may serve as a form of “comfort eating” for those with high punishment sensitivity. This is perhaps not surprising as punishment sensitivity has been associated with negative affect [24], and negative mood has been implicated as a predisposing factor for binge eating [25]. It is possible that some individuals binge eat due to greater reward sensitivity and others due to heightened punishment sensitivity. Evidence of this comes from a study in a nonclinical sample of adolescents, which revealed associations between reward sensitivity and external eating (i.e., eating in response to food cues) and between punishment sensitivity and emotional eating [12].

In addition to binge eating, the present study observed an association between reward sensitivity and compensatory behaviors in women. This finding is consistent with an earlier study showing a positive correlation between reward sensitivity and purging frequency [26]. It is possible that women with heightened reward sensitivity are more likely to binge eat, and thus, may be more likely to engage in inappropriate weight control behavior to avoid negative consequences. This same pattern may not be seen in men in part due to a higher drive for thinness reported by women, which may suggest that men do not regret binge eating as much [27]. Notably, drive for thinness is associated with both reward sensitivity and punishment sensitivity [28]. It may be that if thinness is associated with reward (e.g., leads to praise, etc.), individuals with high sensitivity to reward may be more likely to take such actions to achieve or maintain one’s desired appearance. Further, engaging in compensatory behaviors (i.e. exercising or purging) may be directly reinforcing for women sensitive to reward. Finally, it is possible that the frequency of compensatory behaviors reported by males in this sample was too low to detect this effect. The relationship between reward sensitivity and compensatory behaviors in women merits further exploration.

Study limitations include no formal diagnoses of eating disorders. However, in the shift towards using Research Domain Criteria (RDoC), it is valuable to examine underlying psychological mechanisms associated with key behavioral features of clinical diagnoses in nonclinical samples. Use of a nonclinical sample also resulted in low rates of eating disorder behaviors. Future studies may wish to explore sensitivities to reward and punishment among men and women with eating disorder diagnoses. In addition, findings were based on a college-aged sample, which may not generalize to the overall population. However, these constructs are important to study among this demographic, given that the age of eating disorder onset may occur in young adulthood [29]. The study was also limited by the forced-choice paradigm of the SPSRQ, which may have influenced the results. The current study was limited by a reliance on self-report measures and the cross-sectional design, which precludes causal claims between the variables explored.

## Conclusion

The current study explored the contributions of reward sensitivity and punishment sensitivity to key behavioral features of eating disorders in women and men. Engagement in binge eating and compensatory behaviors did not vary significantly by gender, although there was a trend towards a greater proportion of women engaging in compensatory behaviors. Both men and women reported comparable weekly binge eating episodes over the past three months, but women reported significantly more frequent compensatory behaviors than men. Previous studies in undergraduate samples have observed similar rates of binge eating between men and women. This study demonstrates associations between high reward and punishment sensitivities and specific eating disorder behaviors in both males and females. Additional research aimed at further understanding the role of reward sensitivity and punishment sensitivity in the development and maintenance of eating disorders is needed. Individuals who display treatment resistant behavior may be better served by addressing the underlying factors contributing to their disorders.
